# Gold Nanoparticle-Enabled Cancer Therapy: Translational Design Rules for Photothermal–Immunotherapy and Multimodal Precision Oncology

**DOI:** 10.3390/pharmaceutics18101309

**Published:** 2026-10-15

**Authors:** Xianghua Ma, Wen Xu, Chuda Chittasupho, Dian Zhang

**Affiliations:** 1 Jiangsu Hengrui Pharmaceuticals Co., Ltd., Lianyungang 222007, China; 2 Department of Microbiology, School of Basic Medical Sciences, Xi’an Medical University, Xi’an 710021, China; 3 Department of Pharmaceutical Sciences, Faculty of Pharmacy, Chiang Mai University, Chiang Mai 50200, Thailand; 4 Department of Immunology, School of Basic Medical Sciences, Xi’an Medical University, Xi’an 710021, China

**Keywords:** gold nanoparticles, cancer nanomedicine, photothermal therapy, tumor immunotherapy, drug delivery, radiosensitization, tumor microenvironment, clinical translation

## Abstract

Gold nanoparticles (AuNPs) are versatile cancer-therapy platforms with tunable plasmonic properties, high atomic number, accessible surface chemistry, and potential for integration into theranostic systems. By controlling size, morphology, surface composition, and cargo-loading strategies, AuNPs can support photothermal therapy (PTT), chemotherapy delivery, nucleic-acid transport, radiosensitization, photodynamic therapy (PDT), sonodynamic therapy (SDT), and image-guided treatment. Among these applications, AuNP-mediated PTT is particularly attractive because localized hyperthermia can induce tumor-cell injury, support controlled cargo release, and, under appropriate conditions, contribute to immunogenic cell death and remodeling of the tumor immune microenvironment. However, promising preclinical findings have not translated into widespread routine oncology practice. Major barriers include heterogeneous tumor accumulation, limited penetration beyond perivascular regions, mononuclear phagocyte system uptake, uncertain long-term biodistribution and clearance, inadequate manufacturing reproducibility, and incomplete safety standardization. The enhanced permeability and retention effect is variable across tumors, lesions, and patients; it should not be regarded as a universal basis for clinical nanoparticle delivery. This review summarizes the physicochemical design principles governing AuNP biological performance and critically evaluates their major therapeutic uses in oncology. Particular emphasis is placed on photothermal–immunotherapy, tumor-microenvironment modulation, multimodal synergy, and pharmacokinetic, toxicological, manufacturing, and regulatory barriers. We propose a translational framework connecting material quality attributes, route of administration and in vivo fate, local energy delivery and thermal dosimetry, immune activation, patient selection, and clinically meaningful endpoints. This framework may guide development of more reproducible, controllable, and clinically relevant AuNP cancer therapies.

## 1. Introduction

Cancer treatment relies on surgery, chemotherapy, radiotherapy, molecularly targeted therapy, and immunotherapy. However, these modalities may be limited by systemic toxicity, inadequate tumor selectivity, drug resistance, radiation injury to normal tissues, tumor heterogeneity, and an immunosuppressive tumor microenvironment (TME). Although immune checkpoint inhibitors have improved outcomes in selected malignancies, durable responses remain limited to a subset of patients because of low tumor immunogenicity, poor T-cell infiltration, immune exclusion, and adaptive immune resistance [1,2,3,4].

Nanomedicine offers opportunities to improve the spatial and temporal control of anticancer therapy. Among inorganic nanomaterials, AuNPs have attracted sustained interest because of their shape- and size-dependent optical properties, high atomic number, accessible surface chemistry, and structural diversity [5,6,7,8,9]. AuNPs can be synthesized as nanospheres, nanorods, nanoshells, nanocages, nanostars, nanourchins, ultrasmall nanoclusters, and hybrid architectures. These designs differ in optical absorption, cargo capacity, colloidal stability, cellular uptake, biodistribution, and suitability for activation by external energy [6,7,8,9,10,11].

AuNPs are not merely passive drug carriers. They can function as plasmonic photothermal transducers, radiosensitizers, delivery vehicles for small molecules and nucleic acids, imaging enhancers, and theranostic platforms [5,7,8,9,10,11]. In particular, AuNP-mediated PTT converts absorbed near-infrared light into localized heat. Depending on the thermal dose and tumor context, PTT may damage tumor cells, alter local transport properties, and promote release of tumor antigens and damage-associated molecular patterns (DAMPs) associated with immunogenic cell death [10,11,12,13,14].

Nonetheless, strong in vitro and small-animal findings do not necessarily predict clinical benefit. Systemically administered nanoparticles often show heterogeneous tumor delivery and substantial sequestration in the liver and spleen [15,16,17,18,19]. The enhanced permeability and retention effect is context-dependent, while active targeting mainly improves cell recognition after extravasation and does not guarantee sufficient delivery from the circulation [20,21,22]. Therefore, AuNP development should move beyond generic “tumor-targeting” claims and prioritize clinically controllable systems with defined material quality attributes, predictable pharmacokinetics, measurable local exposure, quantitative treatment control, and rational patient selection.

This disconnect is not merely a translational lag; it reflects a fundamental mismatch between the physicochemical metrics used to optimize AuNPs in vitro (e.g., photothermal conversion efficiency, zeta potential, drug-loading capacity) and the biological variables that determine clinical outcomes (e.g., intratumoral distribution heterogeneity, immune-context-dependent thermal thresholds, and patient-specific EPR variability). Bridging this gap requires explicitly coupling material design to quantitative in vivo fate mapping and clinically interpretable dosimetry.

Recent reviews have comprehensively discussed AuNP material design, photothermal and photodynamic applications, theranostic platforms, and translational barriers [10,11,19,23,24]. The present review does not seek to replace these broad overviews. Instead, it integrates material quality attributes, in vivo fate, treatment dosimetry, immune activity, patient selection, and clinically meaningful endpoints into a practical translational framework. Specifically, we organize these domains as an interdependent development pathway: (i) batch-releasable material quality attributes; (ii) quantitative or spatially resolved in vivo fate assessment; (iii) clinically interpretable thermal-dosimetry metrics, including cumulative equivalent minutes at 43 °C (CEM43 °C) [25]; (iv) functional immune-activation endpoints; (v) biomarker-guided patient selection; and (vi) clinically and regulatory relevant endpoints. The purpose is to identify measurable translational questions and potential go/no-go criteria at each stage, rather than to imply that these individual concepts are entirely new. A comparison with selected recent reviews is provided in Table 1.

This review focuses on translational design rules for AuNP-based cancer therapy, particularly photothermal–immunotherapy and rational multimodal combinations. We propose a practical development framework linking particle properties, biological fate, thermal dose, immune activation, patient selection, and therapeutic endpoints. This review does not aim to catalog all AuNP-enabled anticancer applications. Instead, it uses major therapeutic modalities as case studies to identify the material, exposure, activation, immune, and clinical-design constraints that govern translational feasibility, with particular emphasis on PTT–immunotherapy.

## 2. Physicochemical Design Principles of Aunps

### 2.1. Size-Dependent Biological Behavior

Particle size influences protein adsorption, circulation time, cellular internalization, intratumoral penetration, mononuclear phagocyte system uptake, and elimination route [26,27,28,29]. Ultrasmall gold nanostructures may have greater potential for renal elimination, whereas larger particles can show longer circulation but frequently accumulate to a greater extent in the liver and spleen [26,27,28]. This relationship is non-linear: particles that are too small may be rapidly cleared before adequate tumor exposure, whereas larger structures can remain restricted to perivascular regions.

No universal optimal AuNP diameter exists. Size should be selected according to administration route, target tissue, desired circulation time, clearance requirements, optical function, cargo capacity, and therapeutic mechanism [26,27,28,29]. For example, intratumorally administered PTT agents may prioritize local retention and heat generation, whereas intravenously administered systems must balance circulation, tumor access, tissue penetration, and elimination.

### 2.2. Shape and Plasmonic Properties

AuNP morphology strongly influences localized surface plasmon resonance and photothermal behavior [6,7,8,9,10,11]. Spherical AuNPs typically exhibit plasmon absorption in the visible range, whereas anisotropic structures—including nanorods, nanoshells, nanocages, nanostars, and nanourchins—can be engineered for near-infrared absorption [7,8,9,10,11]. Near-infrared irradiation is generally preferred in PTT because it penetrates tissue better than visible light.

Gold nanorods are widely studied because their longitudinal plasmon resonance can be tuned by aspect ratio. Nanoshells, nanocages, and branched structures can also provide strong near-infrared absorption and photothermal conversion [7,8,9,10,11]. Nanostars and nanourchins generate intense electromagnetic fields near sharp structural features and may support both PTT and surface-enhanced Raman scattering imaging [11]. However, increased structural complexity can complicate synthesis, purification, scale-up, colloidal stability, batch consistency, and regulatory characterization [16,17,18,19].

### 2.3. Surface Chemistry and Protein Corona

Following systemic administration, AuNPs rapidly bind plasma proteins and other biomolecules, forming a dynamic protein corona [30,31,32]. The acquired corona may alter effective particle size, surface charge, colloidal stability, immune recognition, cellular uptake, organ distribution, and clearance [30,31,32]. Thus, material characterization in simple buffer conditions cannot adequately predict in vivo performance.

PEGylation is frequently used to enhance colloidal stability and reduce rapid clearance. However, dense PEG layers may reduce cellular interactions and ligand accessibility, while anti-PEG antibodies can affect the pharmacokinetics of repeatedly administered formulations [33,34,35]. Zwitterionic ligands, biomimetic coatings, cell-membrane camouflage, and tumor-responsive surface layers are potential alternatives. Longer circulation is not always better; dense PEG layers, for example, can suppress the very cellular interactions that therapeutic efficacy depends on.

### 2.4. Active Targeting and Microenvironment Responsiveness

AuNPs can be modified with antibodies, antibody fragments, peptides, aptamers, folate, hyaluronic acid, and other receptor-binding ligands [21,36]. These ligands can increase cellular recognition and internalization after nanoparticles reach the tumor. However, active targeting does not fully overcome barriers related to vascular access, stromal density, elevated interstitial pressure, and heterogeneous perfusion [20,21,37,38].

Tumor-microenvironment-responsive approaches are complementary. AuNP systems can be designed to release cargos or change their behavior in response to acidic pH, elevated glutathione, reactive oxygen species, matrix metalloproteinases, hypoxia, near-infrared irradiation, or ultrasound [39,40,41]. These strategies may improve the therapeutic index by restricting payload activation to tumor tissue rather than relying only on total nanoparticle accumulation.

Although stimulus-responsive delivery concepts are also widely explored in non-gold nanomaterials, hydrogels, and tissue-engineering systems, direct extrapolation to AuNP-enabled cancer therapy should be avoided. In this review, such concepts are considered only when they inform formulation-specific control of AuNP exposure, cargo release, external-energy activation, safety, or manufacturability.

### 2.5. Influence of Synthesis Route and Stabilizers on Biological Performance

The synthesis route is not merely a manufacturing variable; it helps determine the physicochemical and biological identity of an AuNP formulation. Chemical reduction, seed-mediated growth, physical approaches such as laser ablation, and biologically assisted (“green”) syntheses can yield particles with different size distributions, crystallinity, morphology, surface-bound species, residual reagents, and colloidal stability. These features subsequently influence protein-corona composition, cellular uptake, innate immune recognition, biodistribution, photothermal performance, and toxicity. Consequently, two formulations with the same nominal core diameter and shape should not be assumed biologically equivalent when produced via different synthetic routes or purification processes [42,43].

For example, citrate reduction commonly produces negatively charged, citrate-capped spherical AuNPs, whereas seed-mediated syntheses of anisotropic structures, particularly gold nanorods and some branched particles, often use cetyltrimethylammonium bromide (CTAB). CTAB is useful for directing anisotropic growth and maintaining colloidal dispersion during synthesis, but residual free or weakly associated CTAB can disrupt cell membranes and confound interpretation of cytotoxicity, immune activation, and therapeutic effects [42,44]. Thus, for CTAB-derived constructs, rigorous purification and quantitative residual-surfactant testing are essential. Ligand exchange or overcoating with PEG, phospholipids, silica, proteins, polyelectrolytes, or other biocompatible layers may improve physiological stability, but these modifications also alter hydrodynamic size, surface accessibility, cellular internalization, intracellular trafficking, and clearance.

The stabilizer itself can therefore function as a pharmacologically relevant component of the product. Citrate provides relatively weak electrostatic stabilization and may be displaced or masked rapidly in biological media. PEG generally provides steric stabilization and can reduce aggregation and opsonization. However, excessive PEG density may reduce cellular interactions and ligand accessibility, and anti-PEG antibodies may alter the pharmacokinetics or repeat-dose disposition of some PEGylated formulations [35,45]. Biomimetic coatings and green-synthesis-derived coronas may improve compatibility in some settings, but their compositional variability can complicate lot release, mechanistic attribution, and regulatory control. Biologically assisted or green-synthesis approaches may improve apparent compatibility in some systems, but the contribution of natural reducing or capping agents should be distinguished from that of the gold core. Accordingly, vehicle, coating-only, and matched-core controls should be included wherever feasible to support mechanistic interpretation [45,46].

Accordingly, translational characterization should document not only the nominal synthesis recipe but also precursor and solvent quality, reducing-agent and stabilizer identity, seed-to-growth conditions, reaction kinetics, purification method, residual reagent levels, endotoxin burden, storage conditions, and the particle state in relevant biological media. At minimum, measure particle size/PDI, morphology, optical spectrum, zeta potential, hydrodynamic diameter, aggregation behavior, surface-ligand density, and stability in serum-containing media alongside functional assays. For therapeutic studies, comparing otherwise matched AuNPs with different stabilizers can clarify whether observed efficacy or toxicity arises from the gold architecture, the surface chemistry, or their interaction with the protein corona. This synthesis-to-biology linkage should be treated as a critical quality and mechanistic requirement rather than as a supplementary materials issue [47,48]. Morphology-controlled synthesis illustrates this synthesis-to-function relationship. Small changes in reducer concentration, growth time, seed-mediated growth conditions, or purification procedures can alter arm length, aspect ratio, branching, surface-bound species, and plasmonic response. These changes can subsequently influence heat generation, cellular interactions, and therapeutic effects even when nominal gold content is similar. Section 3.1 discusses representative examples of morphology-dependent photothermal behavior.

### 2.6. Recent Advances: Ultrasmall Clusters, DNA-Origami Assembly, and AI-Guided Design

Recent studies have expanded the design space of Au-based nanomaterials and introduced emerging approaches that may support future translational development.

Ultrasmall gold nanoclusters (AuNCs), when engineered to achieve hydrodynamic dimensions compatible with renal elimination, have shown greater renal clearance than larger AuNPs in several preclinical studies. However, this behavior remains strongly dependent on ligand shell, protein interactions, dose, aggregation state, and the effective hydrodynamic size in biological media. For selected ultrasmall formulations, appreciable urinary excretion has been reported within the first 24 h after administration; however, the extent of excretion varies substantially with ligand shell, effective hydrodynamic size, protein interactions, dose, aggregation state, route of administration, and analytical method [49,50]. These clusters exhibit size-dependent fluorescence and can be functionalized with targeting ligands [51]. A renal-clearance-oriented design may help address chronic organ-retention concerns, but it can also reduce systemic exposure and tumor delivery. Each formulation must therefore demonstrate whether active ligands can preserve therapeutically meaningful tumor exposure without compromising renal elimination. Because ultrasmall clusters generally lack the strong collective plasmonic absorption of larger nanostructures, their utility as stand-alone photothermal transducers may be more limited in many settings. They may therefore be particularly attractive for imaging, radiotherapy-related applications, or targeted-delivery functions, although their suitability for PTT should be assessed on a formulation-specific basis.

DNA-origami-templated AuNP systems have been developed for applications including targeted delivery and enzymatic cascade reactions for cancer therapy. A DNA-origami-based enzymatic cascade nanoreactor has been reported to promote chemodynamic cancer therapy and antitumor immune activation in preclinical models [52]. More broadly, DNA-origami platforms may enable spatial organization of inorganic nanocomponents and multifunctional delivery, although the composition, biological activity, and translational feasibility of each construct must be evaluated individually [52,53,54,55]. However, these platforms often integrate multiple functional components, such as targeting aptamers and endosomal-escape peptides, while also facing substantial challenges related to cost, nuclease susceptibility, formulation complexity, and large-scale GMP manufacturing [53,54,55].

AI-guided particle design: Artificial intelligence and machine-learning approaches are increasingly being explored for candidate prioritization, nanoparticle synthesis optimization, process automation, and prediction of selected physicochemical or biological properties in cancer nanomedicine [56,57]. However, prospective validation of models that specifically predict AuNP protein-corona composition, in vivo fate, or therapeutic response remains limited. These computational approaches are emerging tools for candidate prioritization and experimental design, but prospective validation in AuNP development remains early.

Translational takeaway: AuNP physicochemical properties should be treated as product-defining quality attributes only when they can be linked to in vivo fate, local activation, and safety rather than optimized in isolation.

## 3. Aunp-Enabled Therapeutic Modalities and Translational Constraints

### 3.1. Photothermal Therapy

PTT is a defining application of plasmonic AuNPs. Upon irradiation at a wavelength matched to their optical absorption, AuNPs convert light energy into heat through non-radiative relaxation [7,8,9,10,58]. Localized heating can cause protein denaturation, membrane damage, mitochondrial dysfunction, cytoskeletal injury, vascular disruption, and irreversible tumor-cell death [10,11,58,59,60].

Therapeutic outcomes depend on local AuNP concentration and spatial distribution, laser wavelength, fluence, power density, irradiation duration, tumor depth, blood perfusion, and thermal diffusion [10,58,59,60]. Mild or sub-ablative hyperthermia may, in some settings, transiently improve membrane permeability, local perfusion, drug transport, and sensitivity to chemotherapy or immunotherapy. Excessive or spatially uncontrolled heating can injure adjacent normal tissue and alter stromal and immune-cell compartments required for productive antitumor immunity [12,58,59,60].

Recent reviews by Amaral et al. and Obalola et al. show that the field has progressed from plasmonic heat generation to integrated theranostic and combination-treatment strategies; they also emphasize unresolved challenges in biodistribution, reproducible thermal dosimetry, long-term safety, and clinical implementation [10,11]. Photoacoustic imaging, thermal mapping, and other image-guided strategies may support quantitative treatment planning and response assessment [61].

The importance of morphology-controlled synthesis is illustrated by studies from Depciuch and colleagues. In one study, gold nanostars with comparable core dimensions but progressively longer and broader arms were generated by changing the reducer concentration. Increasing arm dimensions shifted the plasmon-resonance maximum from approximately 640 to 770 nm and increased solution heating and calculated photothermal conversion efficiency. In SW480 and SW620 colon-cancer cells, the nanostars with the longest arms produced the greatest loss of viability after 650- or 808-nm irradiation, while morphology-dependent baseline cytotoxicity also required consideration. These findings show that structural features created during synthesis—rather than gold mass alone—govern the optical response, heat generation, and cell-level therapeutic window [62].

A related time-resolved study by the same group showed that terminating a single synthesis at different time points yielded shapes ranging from small spheres to cubic, rod-like, and bone-shaped AuNPs. The resulting particles displayed distinct optical and photothermal behavior, and their effects in irradiated SW480 and SW620 cells varied with synthesis time and morphology. Together, these reports reinforce a practical design principle: shape must be evaluated jointly with surface composition, optical matching to the irradiation wavelength, intrinsic cytotoxicity, and batch-to-batch reproducibility; it should not be reduced to a nominal category such as “nanostar” or “nanorod” [63].

Yang et al. reviewed shape effects in plasmonic photothermal cancer therapy, emphasizing that spherical, rod-like, shell-like, cage-like, and branched architectures differ in their absorption-to-scattering balance, near-infrared resonance, local-field enhancement, heat-generation efficiency, cellular interactions, and suitability for in vivo use [64]. Although anisotropic and branched architectures can provide stronger near-infrared absorption in many settings, this potential advantage must be balanced against stricter synthesis control, structural stability during storage and irradiation, and scale-up complexity. For clinically oriented PTT, evaluation should extend beyond peak temperature rise or photothermal conversion efficiency measured in water to include wavelength-matched optical absorption, performance in protein-containing media and tissue-relevant phantoms, intratumoral spatial coverage, thermal-dose distribution, and normal-tissue heating [58,59,60,61].

### 3.2. Chemotherapy Delivery

AuNPs can carry cytotoxic agents such as doxorubicin, paclitaxel, cisplatin, gemcitabine, and camptothecin derivatives [5,65,66,67,68]. Cargo loading may be achieved through physical adsorption, hybrid nanoassembly, covalent conjugation, or cleavable linkers. Acid-sensitive, redox-sensitive, enzyme-sensitive, and thermoresponsive systems have been investigated to minimize premature drug release and promote local activation [39,40,41,65,66,67].

Combining PTT and chemotherapy is attractive because local heating may facilitate thermoresponsive cargo release, increase membrane permeability, and sensitize tumor cells to cytotoxic injury [5,10,11,65,66,67]. However, as discussed in Section 6.2, total tumor gold content alone does not establish effective drug delivery; rigorous studies should quantify released free drug, intracellular drug exposure, pharmacokinetics, tumor-to-normal tissue ratios, and pharmacodynamic effects. For this review, chemotherapy delivery is considered in the context of PTT–chemotherapy combinations in which local heating can enhance drug release, membrane permeability, and tumor sensitization. However, therapeutic benefit remains agent- and regimen-dependent, and many preclinical studies report additive rather than synergistic effects (Table 2).

### 3.3. Nucleic-Acid Delivery

This section summarizes representative payload classes to illustrate the range of nucleic-acid delivery strategies; detailed mechanistic reviews of each class are available elsewhere. The gold surface permits the high-density presentation of thiol-modified oligonucleotides, enabling the construction of spherical nucleic acids and related nucleic-acid nanoconjugates [74,75,76,77]. Early AuNP platforms were developed mainly for siRNA, antisense oligonucleotides, plasmid DNA, and other short nucleic-acid cargos, for which multivalent surface display can improve nuclease resistance and cell-surface interactions [74,75,76,77,78].

The same design principle has subsequently been extended to additional payload classes, but the delivery constraints differ substantially among them. AuNP–mRNA conjugates and AuNP-containing hybrid carriers have been used to promote intracellular mRNA delivery and expression [79]. In immunotherapy-oriented systems, CpG oligonucleotides conjugated to AuNPs have enhanced innate immune activation and antitumor responses in preclinical models [80]. Gold-based CRISPR delivery platforms have also enabled non-viral co-delivery of Cas9 ribonucleoprotein and donor DNA, providing proof-of-concept for in vivo genome editing [81]. These examples should not be regarded as interchangeable: the requirements for intracellular release, endosomal escape, nuclear access, and functional activity are different for RNA interference, mRNA translation, immune stimulation, and genome editing.

In oncology, AuNP-mediated nucleic-acid delivery has been investigated for oncogene silencing, reversal of drug resistance, immune modulation, and delivery of immunostimulatory cargos [5,74,75,76,77,78,80]. Its performance should be judged by functional activity in the relevant target tissue, for example, target-gene suppression, protein expression, immune activation, or editing efficiency rather than by cellular uptake or total tumor gold content alone (see Section 6.2). Persistent barriers include endosomal sequestration, off-target uptake, heterogeneous intratumoral distribution, innate immune sensing, and manufacturing complexity. Within the present translational framework, nucleic-acid delivery is most relevant when it provides a defined enabling function for PTT–immunotherapy, such as intratumoral immune priming, checkpoint modulation, STING-pathway activation, or sensitization to local thermal injury. While the examples above illustrate the breadth of AuNP-mediated nucleic-acid delivery, their clinical translation will depend on formulation-specific delivery efficiency, safety, and manufacturing feasibility, as discussed in Section 5 and Section 6.

### 3.4. Radiosensitization

The mechanisms and outcomes of AuNP-mediated radiosensitization differ substantially between photon and proton therapies, reflecting their distinct radiation physics and dose deposition profiles. In both contexts, biological outcome depends on nanoparticle localization, intracellular trafficking, radiation quality, oxygenation, and the relative contributions of physical, chemical, and cellular mechanisms. Therefore, radiosensitization cannot be inferred from tumor gold content alone [82].

#### 3.4.1. Photon Radiotherapy: X-Rays and Gamma Rays

For photon irradiation, especially kilovoltage X-rays, gold’s high atomic number (Z = 79) can increase photoelectric interactions relative to soft tissue. Photoelectric absorption produces photoelectrons and subsequent relaxation cascades, including Auger electrons and characteristic X-rays. These short-range secondary emissions can generate highly localized energy deposition around AuNPs; the magnitude and nanoscale distribution of this effect depend strongly on photon energy, particle size and aggregation state, and intracellular localization. The high-Z physical advantage is generally most pronounced at kilovoltage energies, whereas at megavoltage clinical photon energies, Compton interactions predominate and purely macroscopic physical dose enhancement is expected to be smaller [82,83,84].

Observed enhancement under clinically relevant photon conditions is therefore likely to be multifactorial. Proposed contributions include secondary-electron production, increased reactive oxygen species generation, mitochondrial dysfunction, oxidative stress, cell-cycle redistribution, impaired DNA-damage repair, and amplification of DNA double-strand breaks [82,83]. Surface chemistry, particle aggregation, endosomal versus cytosolic localization, proximity to the nucleus, and tumor oxygenation can further influence the outcome. Studies should report photon spectrum or beam quality, delivered dose and fractionation, nanoparticle uptake and subcellular localization, clonogenic survival, DNA-damage endpoints, ROS measurements, and normal-cell controls. Where feasible, dosimetric modeling should be integrated with experimental data to distinguish physical dose enhancement from chemical and biological radiosensitization [82,83].

#### 3.4.2. Proton Therapy

Proton therapy has a distinct rationale because protons deposit energy along a finite path and produce a Bragg peak near the end of their range, thereby allowing conformal dose delivery with reduced exit dose. The interaction of protons with gold does not rely on the same photoelectric mechanism that underpins much of the kilovoltage-photon rationale. Instead, proposed mechanisms include AuNP ionization and secondary-electron emission, localized nanoscale energy deposition, excitation processes and Coulomb-nanoradiator effects, and enhanced aqueous-radical/ROS chemistry [83]. Changes in proton energy and linear energy transfer (LET) across the spread-out Bragg peak further complicate direct extrapolation from photon-radiotherapy studies.

Preclinical evidence supports AuNPs’ potential to enhance proton effects, while also showing the need for cautious, mechanism-specific interpretation. In a proof-of-principle study, CHO-K1 cells were exposed to 10 µg/mL of 50-nm citrate-stabilized AuNPs for 4 h before irradiation with a clinically relevant 200-MeV proton beam. Cunningham et al. reported increased cell killing of 27.1% at 2 Gy and 43.8% at 6 Gy relative to proton irradiation alone, together with enhanced cytogenetic damage at multiple positions along the spread-out Bragg peak [85]. The AuNPs were distributed throughout the cytoplasm, with some particles localized near the nuclear membrane. However, sensitization did not increase monotonically with LET, confirming that no single universal enhancement factor can capture the interplay of beam position, particle distribution, subcellular localization, and biological processes [85].

For proton–AuNP systems, experimental and translational studies should specify initial beam energy, position on the depth-dose curve or within the spread-out Bragg peak, dose-averaged LET, nanoparticle size and coating, intracellular localization, oxygen conditions, and biologically meaningful endpoints such as clonogenic survival and complex DNA damage. Monte Carlo modeling at nano- and cellular scales should be integrated with measured biological data [86]. Key unresolved issues include the relative magnitude of physical versus chemical/biological enhancement at clinical proton energies, the relevance of AuNP clustering and subcellular localization, and whether a sensitization strategy can preserve proton therapy’s normal-tissue-sparing advantage in heterogeneous human tumors [85,86].

### 3.5. Photodynamic and Sonodynamic Combinations

AuNPs can support photodynamic therapy (PDT) through several nonexclusive roles, including photosensitizer delivery, plasmonic enhancement of local electromagnetic fields, incorporation into hybrid photoactive structures, and heat generation in combined PTT–PDT regimens [11]. Depending on the formulation, these systems may concentrate photosensitizers within tumors and modify local excitation conditions, thereby supporting concurrent or sequential oxidative and thermal injury [11,66,67].

However, synergy should not be presumed simply because two modalities are co-formulated. PDT efficacy depends on photosensitizer localization, excitation wavelength, light fluence and fluence rate, oxygen availability, and photobleaching, while PTT depends on AuNP distribution and local thermal dose. Hyperthermia can, depending on dose and timing, enhance photosensitizer uptake, tumor perfusion, and oxidative injury, but excessive vascular damage or oxygen consumption may worsen hypoxia and diminish PDT. Rigorous studies should include matched PTT-only, PDT-only, photosensitizer-only, AuNP-only, light-only, and combination controls; quantify temperature-time profiles and ROS; and distinguish additive from supra-additive effects using prespecified combination analyses. For deeper lesions, limited penetration of excitation light remains a major practical constraint, motivating interstitial fibers, endoscopic delivery, second near-infrared-window strategies, and image-guided treatment planning [66,67].

Sonodynamic therapy (SDT) is attractive because focused ultrasound can reach substantially deeper tissue than externally applied near-infrared light. In AuNP-containing SDT systems, the gold component may serve as a carrier for conventional sonosensitizers, an interface that modifies cavitation-related phenomena, a catalytic or redox-active element, a source of local heating, or a microenvironment-responsive module. Depending on the platform, ultrasound may promote reactive-species formation, acoustic cavitation, mechanical membrane damage, sonoluminescence-associated activation of a co-loaded sensitizer, or enhanced intratumoral transport. The precise role of gold in such composite systems should be established experimentally rather than inferred from its presence alone [87].

The principal translational variables for AuNP-assisted SDT include ultrasound frequency, peak negative pressure, duty cycle, pulse duration, total acoustic exposure, focal geometry, tissue attenuation, cavitation regime, temperature rise, and nanoparticle/sonosensitizer localization. Monitor stable and inertial cavitation where feasible, and measure heating to separate a sonodynamic effect from unintended hyperthermia. Because ROS-mediated SDT may be constrained by hypoxia, rational designs may include oxygen-generating or oxygen-carrying components, but each added function must demonstrate a measurable contribution and not compromise manufacturability. In vivo evaluation should quantify tumor and normal-tissue acoustic exposure, biodistribution, vascular effects, immune consequences, and long-term safety, particularly for repeated treatments [87].

PDT and SDT can also be integrated with immunotherapy when local oxidative or thermal injury yields immunogenic cell-death signals. Nevertheless, inflammatory cytokines alone are insufficient evidence of durable immunity. The same endpoints proposed for PTT–immunotherapy—dendritic-cell activation, antigen-specific T-cell priming, untreated-lesion control, and immune memory—should be used to establish whether multimodal local therapy produces systemic antitumor benefit [12,13,14,77,78].

### 3.6. Comparative Evaluation of Multimodal Strategies

AuNP-enabled multimodal therapy should not be evaluated solely by whether two treatment components can be co-formulated or whether local tumor growth is delayed [69,88,89]. A clinically meaningful comparison should consider at least five dimensions: (i) the strength and reproducibility of local tumor-control data; (ii) evidence for functional systemic antitumor immunity; (iii) dependence on nanoparticle distribution, energy delivery, and treatment sequence; (iv) added safety, manufacturing, and regulatory complexity; and (v) the plausibility of patient selection and quantitative treatment control [10,11,16,17,48].

The comparison below is qualitative rather than a formal meta-analysis. Direct cross-study effect-size comparisons are not scientifically defensible because the published literature differs substantially in tumor model, host immune status, AuNP architecture, route of administration, nanoparticle dose, irradiation wavelength and thermal dose, co-treatment schedule, and endpoint definition [10,11,12,58,59,60]. Therefore, this section and Table 2 identify recurring design strengths, limitations, and translational questions rather than assigning a universal ranking to individual strategies.

Among the strategies considered, photothermal therapy combined with immune checkpoint blockade (PTT–ICB) has comparatively substantial preclinical support for linking local tumor treatment to systemic immune effects in selected immunocompetent models. Mechanistically, PTT may increase tumor-antigen availability and damage-associated molecular pattern signaling, whereas blockade of PD-1, PD-L1, or CTLA-4 can reduce inhibitory constraints on activated T cells [2,12,69]. In bilateral syngeneic models, some optimized regimens have controlled untreated lesions and produced complete responses in a proportion of animals. However, the magnitude and durability of benefit vary markedly with tumor immunogenicity, baseline immune contexture, AuNP formulation and intratumoral distribution, thermal dose, irradiation schedule, checkpoint agent, and endpoint definition [69,70]. These findings should not be interpreted as a generalizable efficacy benchmark. In poorly infiltrated or immunologically cold tumors, PTT–ICB alone may remain insufficient and may require additional immune priming, stromal modulation, or innate-immune-targeted intervention. Studies should therefore include immunocompetent models, untreated-lesion assessment, antigen-specific T-cell endpoints, and, where appropriate, rechallenge experiments to distinguish durable systemic immunity from transient local ablation.

PTT combined with chemotherapy is attractive when local heating can facilitate drug release, increase membrane permeability, or sensitize tumor cells to cytotoxic injury [65,66,67]. This strategy may be particularly useful when the principal objective is local tumor control or when the selected payload has an independently established clinical role. However, the magnitude of benefit is strongly dependent on drug identity, loading and release kinetics, intracellular drug exposure, thermal schedule, and tumor perfusion. Additive rather than demonstrably synergistic effects have been reported in some preclinical settings. Moreover, durable systemic immunity is less consistently demonstrated unless the cytotoxic agent and treatment schedule also induce immunogenic cell-death signals or are combined with an additional immune-modulating component. Translational studies should therefore quantify both local thermal exposure and pharmacologically relevant free-drug exposure in tumor and normal tissues, rather than inferring delivery performance from total tumor gold content alone.

PTT–radiotherapy is mechanistically appealing because AuNPs may influence radiation response while controlled hyperthermia can alter tumor perfusion, permeability, cellular stress responses, and DNA-damage processing [82,83,84,85]. This combination may be most relevant where local control is the primary objective and radiation delivery can be precisely planned. Nevertheless, the interaction depends strongly on radiation modality, beam energy, dose and fractionation, nanoparticle localization, oxygenation, thermal dose, and the sequence of irradiation and heating. For photon radiotherapy, gold’s physical contribution is generally expected to be greater at kilovoltage energies than at clinically common megavoltage energies; for proton therapy, the underlying mechanisms and dose-deposition context differ further. Excessive heating may impair perfusion or worsen hypoxia, potentially offsetting some radiosensitization benefits. Accordingly, studies should integrate nanoparticle localization, radiation dosimetry, spatial thermal-dose mapping, clonogenic survival, DNA-damage endpoints, and normal-tissue exposure rather than relying on tumor-growth delay alone.

PTT combined with STING-pathway activation has a compelling biological rationale because local thermal injury may increase antigen availability and damage-associated signals, whereas STING agonism can promote type I interferon signaling, dendritic-cell activation, and T-cell priming [12,13,69,71]. This approach may be particularly relevant for tumors in which local treatment alone does not generate sufficient innate immune activation. However, the strategy requires careful control of intratumoral co-localization, dose, and timing. Systemic exposure to potent STING agonists can produce cytokine-related toxicity, while nanoparticle co-delivery adds formulation and manufacturing complexity. Direct demonstration of pathway activation, including TBK1/IRF3 phosphorylation and type I interferon-associated readouts, is required; increased inflammatory cytokines alone are insufficient to establish productive STING-dependent antitumor immunity [72,73,89].

PTT–PDT and other photo-oxidative combinations can potentially integrate thermal injury with photosensitizer-mediated reactive oxygen species generation. Such combinations may be useful for accessible tumors or lesions amenable to interstitial, endoscopic, or image-guided light delivery [11,66,67]. However, both modalities depend on spatially controlled energy deposition, and PDT remains sensitive to oxygen availability, photosensitizer localization, photobleaching, excitation wavelength, and light penetration. Hyperthermia may improve drug transport or oxidative injury under some conditions, but excessive vascular injury or oxygen consumption may worsen hypoxia and reduce PDT efficacy. Claims of synergy should therefore be supported by prespecified combination analyses and matched monotherapy controls, including AuNP-only, photosensitizer-only, light-only, PTT-only, PDT-only, and combination groups.

Overall, PTT–ICB currently has relatively stronger preclinical support for systemic immune effects in selected immunocompetent models, whereas PTT–chemotherapy and PTT–radiotherapy may offer more direct routes to local tumor control in selected clinical contexts. PTT–STING and PTT–PDT remain biologically promising but face additional challenges related to local delivery, oxygen dependence, cytokine safety, formulation complexity, and manufacturability. The most appropriate strategy should therefore be selected according to the therapeutic objective, tumor accessibility, route of administration, expected nanoparticle deposition, immune contexture, capacity for real-time treatment monitoring, and feasibility of product-quality control. Table 2 provides a qualitative comparison.

## 4. Aunp-Mediated Photothermal–Immunotherapy

### 4.1. Immunogenic Cell Death

PTT may extend beyond local tumor ablation. Under controlled conditions, heat-induced injury may expose or release tumor antigens and DAMPs, including calreticulin, extracellular ATP, high-mobility group box 1, and heat-shock proteins [12,13,14,69,70,90]. These signals can support dendritic-cell maturation, antigen processing, and tumor-specific T-cell activation.

PTT is not universally immunogenic. Thermal dose, dominant cell-death pathways, tumor type, local immune context, and the timing of combination therapy influence whether local injury produces durable systemic immunity [12,13,14,58,69,70,90]. Studies should therefore assess antigen presentation, dendritic-cell activation, T-cell priming, intratumoral T-cell infiltration, control of distant tumors, and immune memory rather than relying solely on local cytokine changes.

The immunogenicity of PTT is not an intrinsic property of gold nanoparticles or heat exposure; it depends strongly on thermal dose, tumor type, and host immune status.

Thermal-dose dependence. Thermal dose is a major determinant of immunogenic and antitumor outcomes after PTT, but it should not be reduced to a simple threshold. (Table 3) Sub-ablative or mild-to-moderate hyperthermia has been associated with ICD-related signals in some models, whereas rapid high-temperature ablation may produce necrotic injury with less predictable immunogenic consequences [12,13]. These patterns should not be interpreted as universal temperature thresholds, because outcomes depend on cell type, heating kinetics (rate and duration), spatial temperature heterogeneity, local nanoparticle distribution, tumor model, baseline immune contexture, nanoparticle formulation, and combination regimen. Moreover, thermal stress can induce heat-shock-protein release and may be associated with compensatory immunoregulatory changes in some tumor settings; the direction and magnitude of these effects are context-dependent, and in some settings they may contribute to suppression of antitumor immunity [71]. Standardized metrics such as cumulative equivalent minutes at 43 °C (CEM43 °C) can improve reporting consistency, but they do not by themselves predict immunogenicity or capture nanoscale thermal heterogeneity [25].

Tumor-type dependence. In selected immunogenic models, including CT26, nanoparticle-enabled PTT combined with appropriate immune stimulation has induced CD8^+^ T-cell responses and control of untreated distant tumors. In poorly immunogenic tumor settings, PTT alone is often insufficient to generate durable systemic immunity, supporting evaluation of checkpoint blockade, innate immune agonists, or other immune-priming strategies [62,91,92].

Host immune status. In immunocompetent syngeneic models, nanoparticle-enabled PTT combined with immune stimulation or checkpoint blockade can generate durable local and systemic antitumor responses. Demonstration of a durable systemic immune mechanism requires immunocompetent models and functional immune-dependence experiments, because local tumor ablation alone does not establish adaptive antitumor immunity [12,13,14,69,70,90]. In a metastatic melanoma model, Bear et al. showed that combining gold nanoshell-enabled PTT with adoptive T-cell transfer improved local and distant tumor control relative to either treatment alone, illustrating that local thermal ablation may require additional immune engagement to generate robust systemic benefit [93].

Contradictory findings. While some studies report that PTT enhances dendritic-cell maturation and T-cell priming, mild photothermal stress has also been reported to upregulate PD-L1 expression in tumor cells, potentially providing a rationale for combination with immune checkpoint blockade [94]. Local thermal injury may additionally be associated with compensatory immunoregulatory changes, including suppressive myeloid or T-cell responses, depending on the thermal regimen and baseline tumor microenvironment [14]. These apparently divergent findings likely reflect differences in thermal dose, heating kinetics, tumor model, and baseline immune contexture.

**Table 3:** Thermal-Dose Dependence of Immunogenic Cell Death and Antitumor Immunity in AuNP-Mediated PTT.

Thermal Regimen	Temperature/Duration	Cell-Death Modality	ICD Markers (CRT, ATP, HMGB1)	Systemic Immunity (Distant Tumor)	Caveats/Negative Findings
Mild hyperthermia	39–43 °C, 30–60 min	Predominantly apoptosis/ER stress	Elevated in responsive models	Observed in some immunogenic models	Requires functional host immune system; ineffective in T-cell-deficient models
Moderate hyperthermia	43–48 °C, 10–30 min	Mixed apoptosis/necrosis	Variable	Variable across studies	Risk of HSP70-mediated Treg recruitment; PD-L1 upregulation
Ablative thermotherapy	>50 °C, 1–5 min	Predominantly necrosis/coagulation	Not consistently detected under rapid ablative conditions; interpretation is limited by differences in thermal dose, sampling time, tumor model, and assay selection	Reported inconsistently and generally requiring additional immune stimulation or checkpoint blockade	May compromise antigenic epitope integrity; normal-tissue injury risk

Note: The entries summarize patterns reported in selected preclinical studies and are intended as qualitative, context-dependent descriptions rather than universal thermal thresholds. The presence or absence of ICD-associated markers depends on heating kinetics, spatial thermal heterogeneity, tumor type, nanoparticle distribution, sampling time, assay selection, and host immune status. Direct evidence for durable systemic immunity requires assessment of untreated lesions, antigen-specific T-cell responses, immune dependence, and, where appropriate, rechallenge or memory endpoints [12,13,14,69,70,90,93,95,96].

### 4.2. Immune Checkpoint Blockade

Combining PTT with PD-1, PD-L1, or CTLA-4 blockade provides a rational local-to-systemic strategy. PTT may increase local antigen availability and inflammatory signaling, while immune checkpoint blockade can reduce inhibitory constraints on activated T cells [12,13,93,95,96]. This combination may be relevant to poorly infiltrated “cold” tumors, although activity will depend on tumor antigenicity, host immune competence, and the dominant suppressive mechanisms in the TME.

**Figure 1:**
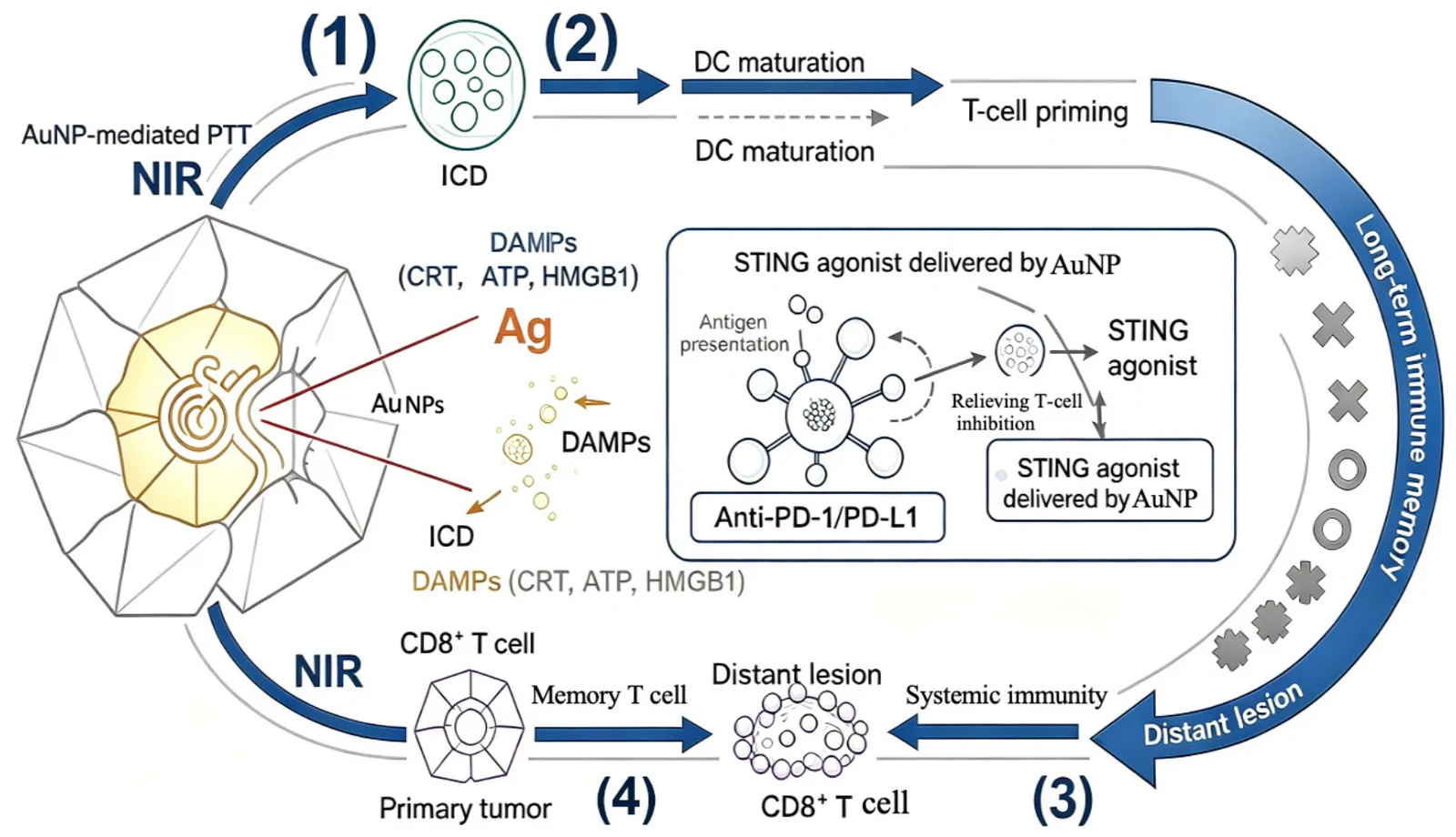
AuNP-mediated photothermal–immunotherapy cycle. Schematic showing: (1) AuNP-mediated PTT induces tumor-cell injury and immunogenic cell death; (2) release of tumor antigens and DAMPs promotes dendritic-cell maturation and T-cell priming; (3) anti-PD-1/PD-L1 therapy or local STING-agonist delivery relieves T-cell inhibition and strengthens innate immune activation; and (4) activated CD8^+^ T cells contribute to control of treated tumors, distant lesions, and immune memory. Abbreviations: AuNP, gold nanoparticle; PTT, photothermal therapy; NIR, near-infrared; ICD, immunogenic cell death; DAMP, damage-associated molecular pattern; CRT, calreticulin; ATP, adenosine triphosphate; HMGB1, high-mobility group box 1; Ag, antigen; DC, dendritic cell; STING, stimulator of interferon genes; PD-1, programmed death 1; PD-L1, programmed death ligand 1; CD8^+^, cluster of differentiation 8-positive.

Preclinical evaluation should include bilateral-tumor or metastatic models, effects on untreated distant lesions, antigen-specific T-cell responses, and, where appropriate, rechallenge experiments [93,95,96]. These endpoints distinguish transient local thermal cytotoxicity from durable systemic antitumor immunity. Figure 1 summarizes the proposed sequence from local photothermal injury to systemic immune activation and memory.

### 4.3. Sting Pathway-Oriented Strategies

The cyclic GMP–AMP synthase–stimulator of interferon genes (cGAS–STING) axis links cytosolic DNA sensing to type I interferon signaling and antitumor immune activation [72,73,89,97]. AuNPs do not inherently activate STING simply because they are gold-based. Instead, they can act as delivery or co-delivery platforms for STING agonists, tumor antigens, nucleic acids, or other immunomodulatory cargos [5,88,98,99]. AuNPs may also be combined with radiation or other DNA-damage-inducing treatments that intersect with innate immune signaling.

While liposomal formulations have demonstrated efficient intratumoral STING agonist delivery [98], an AuNP platform could, in principle, co-localize photothermal injury with intratumoral delivery of a STING agonist. Whether this design improves antitumor immunity relative to sequential or spatially segregated delivery remains unproven.

Clinical experience with intratumoral STING agonists illustrates the need to optimize local exposure, systemic cytokine safety, and combination design; AuNP-based delivery systems may offer one approach to improving these parameters [98,99]. Evidence for STING activation should include direct pathway assessment, such as TBK1 and IRF3 phosphorylation, induction of type I interferon-associated genes, and immune-cell profiling.

### 4.4. Remodeling the Tumor Microenvironment

The TME can limit nanotherapy and immunotherapy through hypoxia, abnormal vasculature, dense extracellular matrix, elevated interstitial pressure, suppressive myeloid populations, regulatory T cells, and impaired antigen presentation [1,2,3,100]. AuNPs can potentially be engineered to deliver immune modulators, oxygen-generating agents, matrix-modifying cargos, macrophage-polarizing agents, or locally activated thermal and oxidative therapies [5,11,100].

The desired endpoint is not merely increased inflammatory markers. A successful strategy should support a functional cancer-immunity cycle that includes antigen release, antigen uptake, dendritic-cell maturation, T-cell priming, tumor infiltration, cytotoxic activity, and immune memory [2,95,100].

Translational takeaway: Evidence of local tumor destruction or inflammatory cytokine release is insufficient; durable photothermal–immunotherapy requires functional systemic immune endpoints including dendritic-cell maturation, antigen-specific T-cell priming, distant-tumor control, and immune memory.

## 5. Challenges in Clinical Translation

### 5.1. Heterogeneous Tumor Delivery

AuNP accumulation depends on vascular density, blood flow, vessel permeability, stromal architecture, extracellular matrix composition, interstitial fluid pressure, lymphatic drainage, lesion size, and immune-cell content [15,20,37,38,101]. Nanoparticle access can vary substantially between and within lesions. Consequently, intravenous administration may produce primarily perivascular localization rather than homogeneous penetration through the entire tumor [37,38], reinforcing that spatially resolved target-site exposure is more informative than bulk tumor gold content (see Section 6.2).

The EPR effect should be viewed as a variable biological phenomenon rather than a universal delivery mechanism [20,37,38]. Clinical development should consider imaging, biopsy-associated biomarkers, or other approaches to identify patients and lesions with sufficient intratumoral nanoparticle exposure. In selected settings, local, intratumoral, intraperitoneal, endoscopic, or image-guided administration may be more appropriate than systemic intravenous delivery.

### 5.2. Biodistribution and Long-Term Safety

After intravenous administration, many AuNP formulations accumulate in the liver and spleen because of mononuclear phagocyte system uptake [16,17,18,19,26,27,28,29]. Long-term safety depends on size, morphology, surface coating, dose, dosing frequency, aggregation state, chemical stability, and clearance route. Larger or persistent gold structures require careful evaluation because short-term animal studies may not capture chronic tissue retention or delayed toxicity.

Safety assessment should extend beyond in vitro viability assays and routine serum chemistry. It should include hemolysis, coagulation, complement activation, cytokine release, immunotoxicity, histopathology, chronic organ accumulation, reproductive toxicity where relevant, and interactions with accompanying cancer therapies [16,17,18,19,48]. The study design should reflect the intended clinical schedule, particularly for repeated dosing and multidrug combinations.

Quantitative chronic-toxicity data are essential for clinical design. Repeated intravenous administration of persistent AuNP formulations can produce measurable and prolonged gold retention in the liver and spleen in rodents. The magnitude varies substantially with core size, hydrodynamic diameter, ligand shell, particle shape, dose, dosing interval, route, and analytical method [102,103]. Ultrasmall formulations designed to maintain an effective hydrodynamic size compatible with renal filtration may show faster urinary elimination than larger plasmonic constructs, but renal clearance must be demonstrated using formulation-specific in vivo mass-balance and recovery data [50,104].

Importantly, nominal core size alone is insufficient to predict renal clearance, because the effective hydrodynamic diameter and protein interactions in biological media may differ substantially from the primary particle size measured in buffer.

Implications for clinical dosing schedules:

- Track cumulative lifetime gold exposure.
- For repeated-dose regimens, justify the dosing interval and follow-up duration with formulation-specific biodistribution and long-term toxicology data; no universal washout interval can currently be recommended.
- For repeat-dose systemic regimens, incorporate hepatic and renal laboratory monitoring into protocol-specific safety plans, with testing frequency determined by the formulation’s biodistribution, clearance pathway, dose, and accompanying anticancer therapies.
- Where justified by the formulation and available imaging sensitivity, early clinical studies may incorporate exploratory imaging or quantitative biodistribution approaches to assess organ retention. Establish the suitability of CT, MRI, photoacoustic imaging, PET, or other methods for each platform.

Table 4 provides an illustrative, formulation-dependent summary of biodistribution and clearance considerations.

### 5.3. Manufacturing and Quality Control

AuNP properties may be altered by relatively small changes in synthesis conditions, precursor purity, seed-mediated growth, residual surfactants, ligand exchange, endotoxin contamination, storage, aggregation, and sterilization methods [16,17,18,19,48,105]. Such variation can affect therapeutic activity and toxicity. The following examples are illustrative development targets rather than universal regulatory acceptance criteria; final specifications should be product-specific, clinically justified, and validated [47,48,105].

A translational AuNP product should specify critical quality attributes, including particle-size distribution, morphology, optical absorption spectrum, surface-ligand density, surface charge, drug loading, free-drug content, colloidal stability, endotoxin burden, sterility, and batch-to-batch consistency [16,17,18,19,48,105]. Manufacturing control is not a secondary technical task; it is essential to reproducible preclinical and clinical interpretation.

Numerical specifications such as a narrow PDI range or inter-batch CV target should be viewed as formulation-specific development criteria rather than universal regulatory thresholds. Sponsors should prospectively justify their acceptability by demonstrating relationships between the critical quality attribute and pharmacokinetics, treatment performance, safety, or clinical use.

In practice, for development-stage comparability studies, sponsors may prospectively define formulation-specific targets—such as a validated PDI range (e.g., ≤0.20 in some systems), morphology acceptance criteria by TEM, a prespecified optical-spectrum window, and batch-to-batch variability limits for CQAs. However, these values are illustrative development targets and are not universal regulatory release specifications. Final acceptance criteria should be product-specific, clinically justified, and validated.

Concrete quality-control metrics and regulatory context: A translational AuNP product should specify critical quality attributes (CQAs), including:

- Particle-size distribution: DLS-derived size distribution and PDI may be prospectively specified within a validated product-specific range. A PDI value of approximately 0.20 may serve as an illustrative development target for selected formulations, but it is not a universal regulatory criterion and may not apply to anisotropic, multimodal, or intentionally polydisperse systems.
- Optical properties: UV–Vis–NIR spectra may be specified within a product-specific acceptance window that reflects the intended morphology, plasmon-resonance position, and photothermal function.
- Surface chemistry: Ligand density should be measured using a formulation-appropriate validated method, such as XPS, TGA, colorimetric analysis, or another suitable assay. Zeta potential may be specified within a product-specific range when it is linked to colloidal stability, protein interactions, or biological performance.
- Safety-related attributes: Endotoxin, sterility, residual surfactants, and residual reagents should be assessed using validated methods appropriate for the intended route of administration. Control residual CTAB or other synthesis-derived species using formulation-specific acceptance criteria justified by relevant toxicological and functional data.

Batch consistency: Predefine inter-batch variability limits and justify them by product. A coefficient of variation below 10% for selected CQAs may serve as an internal development target, supported by process validation and functional comparability data, rather than a universal release specification.

Regulatory guidance: FDA’s April 2022 final guidance for industry, “Drug Products, Including Biological Products, that Contain Nanomaterials,” emphasizes characterization of size distribution, surface properties, and stability. EMA has issued reflection papers addressing nanomedicine characterization requirements, emphasizing the need for case-by-case evaluation of nanoparticle quality attributes, batch-to-batch equivalence, and in vitro–in vivo correlation. ICH Q3D provides guidance on elemental impurities control, which is relevant to potential process-derived metal contaminants; ICH Q6A covers product specifications. These guidelines should be interpreted in the context of AuNP products, where gold itself is the active/pharmaceutical component rather than a contaminant.

Scale-up challenges: (i) Scale-up of seed-mediated growth for anisotropic AuNPs may alter nucleation and growth kinetics, mixing, heat and mass transfer, ligand distribution, and purification performance, thereby changing particle morphology, aspect ratio, optical properties, and batch-to-batch comparability. These effects are formulation- and process-dependent and should be evaluated through scale-down models, process characterization, and predefined comparability criteria rather than by applying a universal numerical threshold [16,17,18,19,42,43,44,45,46,47,48,105]; (ii) ligand-exchange efficiency drops at larger scales due to mass-transport limitations; (iii) sterile filtration of non-spherical AuNPs can alter morphology. Mitigation strategies include continuous-flow microreactor synthesis and in-line DLS monitoring.

### 5.4. Clinical Dosimetry and Treatment Control

For AuNP-mediated PTT, the administered nanoparticle dose alone does not define treatment intensity. A reproducible clinical protocol should specify local AuNP exposure, laser wavelength, power density, irradiation area, exposure duration, local temperature distribution, thermal dose, and the response of adjacent normal tissues [10,58,59,60,61]. Image guidance and real-time thermal monitoring can improve treatment precision and safety.

Photoacoustic imaging, magnetic resonance thermometry, infrared thermal imaging, and computational modeling may facilitate nanoparticle tracking and thermal-dose planning [61,106]. Deeper tumors may require interstitial optical fibers, endoscopic approaches, or ultrasound-enabled hybrid modalities. A clinical pilot study of image-guided focal ablation using intravenously administered silica–gold nanoshells followed by near-infrared laser irradiation in localized prostate cancer provided early evidence of the technical feasibility of focal photothermal intervention; however, standardized protocols, controlled comparative studies, and longer-term oncologic outcome data remain necessary [107].

### 5.5. Clinical Evidence and Barriers to Regulatory Approval

Clinical evidence for gold nanoshell-mediated photothermal ablation remains limited to early-phase and pilot studies. In the clinical pilot study reported by Rastinehad et al. [106], sixteen men with low- or intermediate-risk localized prostate cancer received gold-silica nanoshell infusion followed by MRI–ultrasound fusion-guided laser ablation. Focal ablation was successfully achieved in 15 of 16 patients (94%). Follow-up assessments included multiparametric MRI at 48–72 h and imaging- or biopsy-based evaluations at 3 and 12 months. No treatment-related serious adverse events or clinically meaningful deterioration in urinary or sexual function were reported during the reported follow-up period. However, the study was small, non-randomized, and primarily designed to evaluate feasibility and safety; the observed biopsy findings should therefore not be interpreted as definitive evidence of oncologic efficacy [107].

Two registered AuroLase studies evaluated related applications in refractory or recurrent head-and-neck tumors (NCT00848042) and primary or metastatic lung tumors (NCT01679470). The head-and-neck study was registered as a pilot study with a planned follow-up of up to 6 months and included tumor response and treatment-related adverse-device effects as outcome measures. The lung-tumor study included assessment of thermal-lesion formation at 6 months. As checked on 25 September 2026, the registry pages did not provide posted results sufficient to quantify clinical response or safety outcomes for these studies. The absence of posted results should not be interpreted as evidence of a negative treatment effect. For NCT00848042, the registry records a completed study status, whereas NCT01679470 is listed as terminated; these registry status labels describe trial disposition and do not, by themselves, establish treatment efficacy or inefficacy [108,109].

Clinical experience with other gold-based nanomedicines further illustrates that early human studies can establish feasibility, pharmacokinetics, and acute safety without demonstrating definitive therapeutic benefit. In a phase I dose-escalation study, PEGylated colloidal gold-rhTNF (CYT-6091) was administered to 24 patients with advanced solid tumors. The study did not reach a maximum tolerated dose, reported manageable cytokine-associated toxicity, and was not designed or powered to establish antitumor efficacy; no objective responses were reported [110]. However, experience with a gold-based formulation designed for systemic cytokine delivery cannot be directly extrapolated to gold nanoshell-mediated local photothermal ablation, because nanoparticle architecture, route of administration, treatment mechanism, local energy delivery, biodistribution, and relevant safety risks differ substantially.

Several barriers continue to limit the transition from technical feasibility to clinically meaningful and reproducible benefit. First, local treatment effect depends on sufficient and spatially appropriate nanoparticle deposition, which is affected by lesion perfusion, vascular permeability, stromal structure, administration route, and intratumoral heterogeneity [16,17,18,19]. Second, administered particle dose alone does not define photothermal treatment intensity. Clinical protocols should report the local nanoparticle exposure, irradiation wavelength, fluence, power density, irradiation area, exposure duration, temperature–time profile, thermal-dose distribution, and normal-tissue heating [58,59,60,106]. Where feasible, use image guidance and quantitative thermal monitoring to document target-site exposure and treatment delivery. Gold nanoparticles may also support photoacoustic imaging, although the suitability, sensitivity, and clinical utility of any imaging approach must be established for each specific platform and treatment setting [61,106].

Third, acute procedural safety does not establish long-term systemic safety. Depending on particle size, hydrodynamic diameter, shape, surface chemistry, dose, dosing frequency, aggregation state, and route of administration, gold nanomaterials may persist in the liver, spleen, or other components of the mononuclear phagocyte system [16,17,18,19]. Long-term follow-up should therefore include formulation-specific assessment of biodistribution, clearance, organ retention, hepatic and renal function, hematologic effects, immunotoxicity, and potential interactions with concomitant anticancer therapies. These requirements may be particularly important for intravenously administered or repeat-dose regimens, whereas locally administered single-treatment strategies may have a different risk–benefit profile.

Finally, regulatory development requires a well-defined and reproducible product rather than a nominal “gold nanoparticle” identity. Product-specific critical quality attributes should include particle-size distribution, morphology, optical spectrum, surface-ligand chemistry and density, aggregation behavior, residual synthesis reagents, sterility, endotoxin burden, and stability under clinically relevant storage and administration conditions. Justify the acceptability of each specification by its relationship to pharmacokinetics, local heat generation, treatment reproducibility, biodistribution, safety, or clinical performance. Because small changes in particle morphology or surface chemistry can influence optical behavior and biological fate, chemistry, manufacturing, and controls should be integrated with preclinical comparability testing and clinical protocol design from the earliest stages of translation [16,17,18,19,47,105].

Taken together, the available clinical evidence supports technical feasibility and short-term procedural safety in selected settings, but it remains insufficient to establish durable tumor control, systemic antitumor immunity, long-term nanoparticle safety, or comparative clinical benefit. Future prospective studies should include prespecified efficacy endpoints, standardized treatment dosimetry, systematic adverse-event reporting, pharmacokinetic and biodistribution assessments, clinically meaningful follow-up duration, and transparent reporting of registered-trial results regardless of outcome [16,17,18,19,47,102,103,104,105,106].

## 6. Translational Development Framework

A successful AuNP system should not be judged only by in vitro photothermal conversion, cell killing, or nominal tumor accumulation. Its development should follow a connected framework: material attributes → in vivo fate → local thermal dose → immune activation → patient selection → clinical endpoints. Table 5 provides a practical checklist for translating AuNP cancer therapeutics.

The design rules below are proposed translational recommendations synthesized from the available preclinical, clinical, and regulatory literature. They are not intended to replace product-specific regulatory guidance or validated clinical-development requirements.

A cross-cutting determinant of all six domains is the clinical delivery context, including route of administration (e.g., intravenous, intratumoral, image-guided), lesion accessibility, and energy-delivery strategy (e.g., external near-infrared irradiation, interstitial fibers, endoscopy-guided delivery). These variables often determine whether a given AuNP formulation can be translated, and they should be evaluated prospectively alongside the six core domains.

### 6.1. Material Quality Attributes

Critical material quality attributes include particle-size distribution, morphology, optical absorption spectrum, surface charge, ligand density, drug or nucleic-acid loading, release kinetics, colloidal stability, sterility, endotoxin burden, and batch-to-batch reproducibility [16,17,18,19,48,105]. These parameters should be measured using validated methods and, wherever possible, linked to functional outcomes such as circulation time, tumor uptake, photothermal conversion, release kinetics, and immune activation.

### 6.2. In Vivo Fate and Target-Site Exposure

Systemic development programs should establish blood pharmacokinetics, tumor exposure, tumor-to-normal tissue ratios, liver and spleen uptake, clearance routes, persistence, and the impact of protein-corona formation [15,16,17,18,19,20,26,27,28,29,30,31,32,37,38]. Importantly, total tumor gold content is not always sufficient. Intratumoral distribution, cellular localization, penetration depth, and co-localization with the relevant therapeutic target may be more informative than bulk accumulation alone.

### 6.3. Thermal Dosimetry

For PTT, describe exposure using measurable physical variables rather than nominal laser settings alone. Relevant parameters include local nanoparticle concentration, irradiation wavelength, fluence, power density, irradiation duration, temperature-time curves, spatial temperature heterogeneity, and normal-tissue heating [10,58,59,60,61]. Where possible, standardize thermal dose using clinically interpretable measures, such as cumulative equivalent minutes at 43 °C, alongside real-time imaging or thermometry [25].

### 6.4. Immunological Activity

For photothermal–immunotherapy, the desired outcome is a coordinated immune response rather than nonspecific inflammation. Preclinical assessment should include immunogenic cell-death markers, dendritic-cell maturation, antigen cross-presentation, tumor-specific CD8^+^ T-cell priming, intratumoral immune-cell composition, regulatory T-cell and myeloid suppressor-cell changes, systemic cytokine profiles, distant-tumor control, and immune memory [2,12,13,14,37,69,70,90,93,95,96].

### 6.5. Patient Selection and Clinical Endpoints

The following variables should be regarded as hypothesis-generating enrichment candidates rather than validated eligibility thresholds for AuNP-based clinical trials. These variables should be incorporated initially as exploratory stratification, pharmacodynamic, or treatment-planning measures in early-phase studies rather than as universal exclusion criteria.

(i) Tumor accessibility: Candidate lesions should be accessible to the selected energy-delivery approach, including external near-infrared irradiation for superficial lesions, endoscopic or interstitial optical access, or intraoperative illumination. Where appropriate, image-guided intratumoral injection may improve local AuNP deposition. Deep visceral lesions beyond the effective penetration range of the selected optical-delivery approach are generally poor candidates for externally applied PTT unless interstitial, endoscopic, intraoperative, or other localized energy-delivery strategies are feasible [58,59,60].

(ii) Vascular permeability/nanoparticle deposition: Imaging metrics such as DCE-MRI Ktrans or photoacoustic signal thresholds may serve as exploratory indicators of tumor perfusion and vascular phenotype; cutoffs should be established and validated for each specific imaging modality, AuNP formulation, and tumor type [15,37,38,61,111]. Where a validated platform-specific imaging or assay method is available, consider confirming intratumoral AuNP exposure as an enrichment or treatment-planning criterion.

(iii) Immune contexture: Baseline CD8^+^ tumor-infiltrating lymphocyte density and PD-L1 status may serve as exploratory immune-phenotyping variables [112,113]. Their predictive value for AuNP-based PTT + ICB responses requires prospective validation and should not be assumed equivalent to their role in checkpoint-blockade monotherapy [114].

(iv) Thermal tolerance margin: Safe thermal dosing requires adequate distance from critical structures. The required margin should be determined by tumor location, thermal conduction modeling, energy-delivery modality, and real-time thermometry capabilities [115]. A fixed distance should not be treated as a universal requirement.

Non-invasive measurement modalities: Photoacoustic imaging and NIR fluorescence (if fluorophore-conjugated) track AuNP deposition; MRI thermometry maps thermal dose in real time [116]. Radiolabeled AuNP constructs, including research platforms labeled with radionuclides such as copper or zirconium isotopes, may enable PET-based quantification of biodistribution and tumor exposure [117]. Their suitability depends on radiolabel stability, isotope half-life, dosimetry, and regulatory feasibility; Baseline tumor biopsy, when feasible, can support assessment of immune contexture through histology, immunohistochemistry, multiplex imaging, or transcriptomic profiling. Liquid-biopsy markers, including ctDNA, may complement tumor-burden and molecular-response monitoring but do not directly replace tissue-based immune profiling [118].

## 7. Future Perspectives

AuNP cancer therapies should be developed through a clinically oriented framework. Nanoparticles should be designed for a defined route of administration, target lesion, treatment-control strategy, and clinical objective rather than optimized only for in vitro photothermal efficiency or nominal tumor accumulation. Incorporate patient stratification early, especially for platforms intended for intravenous administration.

The field should also avoid unnecessary multifunctionality. The temptation to add one more functional layer—targeting ligands, responsive polymers, imaging probes—is understandable, but each addition introduces a new failure mode in manufacturing and a new variable in regulatory review [5,16,17,18,19]. In many cases, simpler systems with measurable pharmacokinetics, a clear mechanism of action, and a specific clinical use case may have a more realistic route to translation.

A near-term clinical opportunity is likely in accessible tumors where nanoparticle deposition and the optical treatment field can both be verified before activation. In this setting, intratumoral or image-guided administration may reduce dependence on heterogeneous systemic delivery. The central translational question is not whether an AuNP construct can generate heat in a tumor, but whether its spatial distribution permits a reproducible thermal dose that can be coupled to a defined immunological intervention and a measurable patient-relevant outcome.

## 8. Conclusions

**Figure 2:**
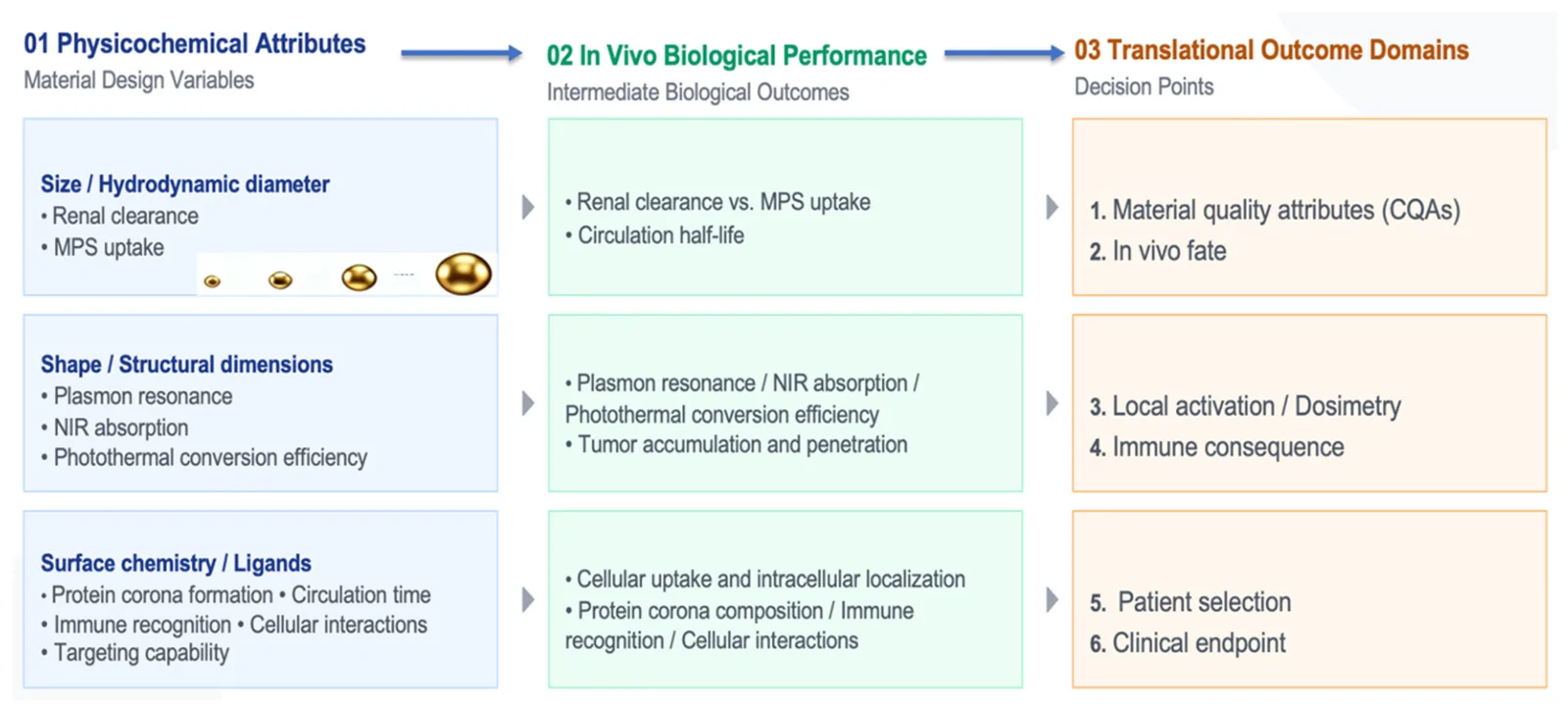
Systematic mapping of AuNP physicochemical attributes to biological and clinical performance metrics. Size, together with hydrodynamic diameter and surface chemistry, strongly influences the balance between renal elimination and MPS uptake; shape and structural dimensions influence plasmon resonance, NIR absorption, and photothermal performance; surface chemistry affects protein-corona formation, circulation, immune recognition, and cellular interactions. Arrows indicate how each attribute feeds into the six-domain translational framework.

Near-term development priorities: Image-guided local PTT for accessible tumors, including selected prostate, head-and-neck, and other lesions amenable to intratumoral, endoscopic, interstitial, intraoperative, or image-guided AuNP delivery. Development programs should incorporate real-time thermal monitoring and predefined go/no-go criteria (Figure 2), including confirmation of adequate intratumoral AuNP deposition before laser activation where technically feasible.

Medium-term development priorities: PTT combined with checkpoint blockade in biologically selected patient populations. Candidate enrichment variables may include accessible lesions, measurable intratumoral exposure, baseline immune phenotype, and feasibility of controlled local thermal delivery. Progression beyond early clinical development should require evidence of treatment-associated immune activation, acceptable safety, and clinically meaningful local or systemic activity.

Longer-term research directions: Systemic photothermal–immunotherapy and integrated multimodal theranostic platforms. These remain aspirational because heterogeneous human tumor delivery, variable EPR-related transport, long-term biodistribution, manufacturing comparability, and regulatory assessment of complex combination products remain unresolved.

## Figures and Tables

**Table 1:** Positioning of the Present Review Relative to Selected Recent Reviews of AuNP-Enabled Cancer Nanomedicine.

Feature	Amaral et al. 2025 [10]	Obalola et al. 2025 [11]	Mitchell et al. 2021 [19]	This Review
Core focus	PTT theranostics	PDT/PTT advances	General nanoparticle delivery	Translational design rules for PTT-immunotherapy
Framework structure	Discussed across separate thematic sections	Discussed across separate thematic sections	Discussed across separate thematic sections	Sequential six-domain chain (material quality attributes → fate → dose → immunity → patient → endpoint)
Thermal dosimetry	Discussed at a general level	Discussed at a general level	Not a primary focus	Standardized metrics (CEM43 °C) linked to ICD
Immune activation	General discussion	General discussion	General discussion	Critical analysis of ICD conditions and model dependence
Patient selection	Not a primary focus	Not a primary focus	Not a primary focus	Biomarker-guided enrichment criteria
Multimodal comparison	Descriptive listing	Descriptive listing	Not a primary focus	Qualitative cross-strategy comparison
Manufacturing/regulatory	Discussed at a general level	Discussed at a general level	Discussed at a general level	Concrete CQAs and regulatory guidance
Clinical trial data	Limited discussion	Limited discussion	Limited discussion	Expanded discussion of selected clinical studies
Long-term toxicity	Addressed to a limited extent	Addressed to a limited extent	Addressed to a limited extent	Expanded discussion of biodistribution and monitoring
Roadmap	Not explicitly integrated as an operational sequence	Not explicitly integrated as an operational sequence	Not explicitly integrated as an operational sequence	Tiered near/mid/long-term roadmap

**Table 2:** Qualitative comparative assessment of AuNP-enabled multimodal strategies.

Strategy	Principal Therapeutic Objective	Evidence for Local Control	Evidence for Systemic Immunity	Main Translational Advantage	Major Limitation/Common False-Positive Interpretation	Relative Translational Readiness
PTT-ICB	Local treatment plus systemic immune priming	Substantial in selected immunocompetent models	Comparatively substantial but model-dependent	Rational local-to-systemic mechanism; immune therapy components may already have clinical pathways	Local ablation or cytokine rise may be misread as durable immunity; activity in cold tumors remains variable	Moderate for accessible tumors with measurable deposition, controlled heating, and biologically selected patients
PTT-chemotherapy	Local tumor control and triggered drug release	Substantial but agent- and schedule-dependent	Usually limited unless combined with immunogenic or immune-modulating therapy	Direct local cytotoxic synergy; may use established drugs	Total tumor gold is not proof of free-drug exposure or synergy	Moderate for local or regional disease if release kinetics and normal-tissue exposure are controlled
PTT-radiotherapy	Local control and radiosensitization	Promising in selected preclinical models but strongly dependent on radiation and thermal parameters	Limited or indirect in most studies	Can integrate with established local radiation platforms	High-Z rationale is often overgeneralized, especially at megavoltage photons; incomplete dosimetry	Low-to-moderate; requires rigorous spatial radiation and thermal dosimetry
PTT-STING agonism	Innate immune priming and T-cell activation	Promising in preclinical systems	Promising but requires direct mechanistic validation	Addresses insufficient innate immune activation after local therapy	Inflammatory cytokines alone do not establish STING-dependent immunity; cytokine toxicity risk	Low-to-moderate; intratumoral co-localization and safety control are essential
PTT-PDT	Combined thermal and oxidative local injury	Promising for accessible lesions	Variable and generally less established	Complementary mechanisms; potential spatial co-activation	Oxygen dependence, limited light penetration, and photosensitizer photobleaching may restrict treatment depth; apparent combination benefit can be misread as synergy without matched monotherapy controls	Low-to-moderate for accessible lesions; requires wavelength-matched dosimetry, oxygen-aware treatment planning, and formulation-specific safety and manufacturing control

Abbreviations: AuNP, gold nanoparticle; PTT, photothermal therapy; ICB, immune checkpoint blockade; PDT, photodynamic therapy; STING, stimulator of interferon genes. Note: Table 2 provides a qualitative evidence-mapping assessment rather than a formal meta-analysis, systematic ranking, or pooled efficacy comparison. “Substantial,” “promising,” “limited,” and the relative translational-readiness categories reflect the type, consistency, and mechanistic depth of the available evidence, together with clinically relevant constraints including route of administration, tumor accessibility, nanoparticle deposition, thermal or radiation dosimetry, immune-context dependence, manufacturing complexity, and safety monitoring. Because the underlying studies differ in nanoparticle composition, dose, tumor model, host immune status, treatment schedule, and endpoint definition, do not interpret the assessments as universal efficacy rankings [10,11,12,13,16,17,18,19,48,58,59,60,69,70,71,72,73].

**Table 4:** Illustrative formulation-dependent patterns of biodistribution and clearance relevant to AuNP translational design.

Size Class	Examples	Commonly Observed Disposition Tendency	Potential MPS-Retention Concern	Typical Translational Concern	Development Implication
Ultrasmall clusters	<3 nm (Au25, Au102)	May favor renal elimination if effective hydrodynamic diameter remains compatible with filtration	Often lower than larger persistent constructs, but formulation-dependent	Rapid	May be more compatible with repeat-dose development, subject to formulation-specific pharmacokinetic and long-term toxicology evaluation
Small spheres	3–6 nm	Renal + hepatobiliary	Moderate	Intermediate	Requires formulation-specific renal and hepatobiliary disposition assessment
Standard theranostic	10–50 nm (nanorods, nanoshells, nanostars)	MPS (liver, spleen)	Frequently associated with increased liver/spleen uptake in preclinical systems	Prolonged	May require long-term biodistribution, hepatic/splenic safety monitoring, and cumulative-exposure justification
Large aggregates	>100 nm	MPS (liver, spleen, lung)	Very high	Potential for prolonged retention; requires formulation-specific assessment	May pose substantial challenges for systemic repeat-dose development

Note: Nominal core size alone does not determine in vivo fate. The categories are conceptual and should not be interpreted as universal pharmacokinetic rules. Hydrodynamic diameter, surface-ligand chemistry, shape, dose, route, protein corona, aggregation state, and species substantially influence clearance and organ retention. Clearance and retention categories are generalized from multiple preclinical studies [26,27,28,29,49,50,102,103,104]; exact values depend on the formulation and should be determined for each product.

**Table 5:** Translational evaluation checklist for AuNP cancer therapeutics.

Development Domain	Core Question	Recommended Measurements	Common Pitfalls/False Positives
Material quality	Is the product reproducible and stable?	Size/PDI, morphology, optical spectrum, ligand density, drug loading, sterility, endotoxin, storage stability	Assuming buffer-phase characterization predicts in vivo corona behavior
Pharmacokinetics and exposure	Does systemic and intratumoral exposure support the intended use?	Blood concentration–time profile, tumor-to-blood ratio, organ distribution, clearance, intratumoral localization	Equating total tumor gold content with functional intratumoral exposure or effective thermal coverage
Treatment control	Is the product reproducible and safe?	Wavelength, fluence, power density, temperature–time profile, thermal dose, normal-tissue temperature	Reporting laser settings or nominal power density without intratumoral temperature mapping, thermal-dose estimation, or normal-tissue exposure
Immune activity	Does the product induce antitumor immunity?	Immunogenic markers, maturation, CD8+ T cells, Treg/MDSC profile, distant-tumor effect, immune memory	Confusing transient inflammation (IL-6 spike) with durable antigen-specific immunity
Safety	Is the product treatment acceptable?	Hemolysis, cytokines, retention, histology, organ function	Focusing on acute toxicity while missing chronic organ retention
Clinical activity	Is there meaningful patient benefit?	Objective response, progression-free survival, local control, surveillance, quality of life, adverse events	Interpreting preliminary response without considering durability, safety, patient-reported outcomes, and appropriateness to the trial phase

## Data Availability

No new data were created or analyzed in this study. Data sharing is not applicable to this article.
